# A Case Report of Secondary Spontaneous Pneumothorax Induced by Vape

**DOI:** 10.7759/cureus.6067

**Published:** 2019-11-04

**Authors:** Munish Sharma, Humayun Anjum, Chinthaka P Bulathsinghala, Mihir Buch, Salim R Surani

**Affiliations:** 1 Internal Medicine, Corpus Christi Medical Center, Corpus Christi, USA; 2 Pulmonary/Critical Care, Corpus Christi Medical Center, Corpus Christi, USA; 3 Internal Medicine, Texas A&M Health Science Center, Temple, USA

**Keywords:** vaping induced lung injury, secondary spontaneous pneumothorax, electronic cigarette, ground glass opacities, tetrahydrocannabinol

## Abstract

Electronic cigarettes (referred here as E-cigarettes or vapes) are devices that contain heated nicotine/cannabinol vaporized aerosol solution for consumption. While long-term toxicities of E-cigarettes are unknown, the acute adverse events of vaping that have occurred are concerning. There have been variations of pneumonitis presentations so far, however, very few case reports have been shown to have a complication of a pneumothorax. We hereby present a case of a 35-year-old male who presented with spontaneous pneumothorax and pneumonitis due to vaping.

## Introduction

Electronic cigarettes (referred here as E-cigarettes) were initially created as devices to deliver a nicotine-containing aerosol to users by heating a solution typically made up of propylene glycol (PG) or glycerol, nicotine, and flavoring agents without agents such as tars, oxidant gases, and carbon monoxide smoke found in traditional tobacco cigarettes [1-2]. E-cigarettes entered the United States in 2006, but its usage has increased significantly since 2010 [3]. Their use has exponentially increased due to the belief that individuals using electronic cigarettes are not exposed to the traditional harmful effects of cigarette smoking. Additionally, their use has also been found to be helpful in cutting down the number of cigarettes smoked to those who have to completely quit smoking and helped individuals remain off cigarettes for a long time [4]. While the toxicities of E-cigarettes in the long term are not fully known, the acute adverse events of vaping that have occurred are concerning. There has been an official count of 450 U.S. cases reported in 33 states and one territory since 9/6/19 with five deaths reported [5]. These patients have presented with various types of pneumonitis. No definite chemical or causation has been found to be the culprit, however, the main components have been nicotine (some have been produced to be nicotine free), PG or glycerol, and flavorings. There has also been an association with cannabinoid oil [6-8]. We present a case of vaping-induced lung injury leading to a secondary spontaneous pneumothorax. 

## Case presentation

**Figure 1:**
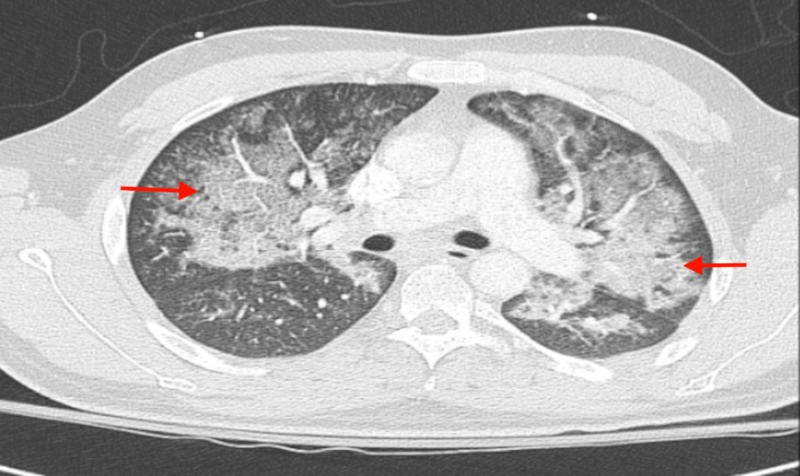
CT angiogram of the chest showing extensive ground glass opacity in both lungs mostly sparing the periphery.

A 35-year-old male with no past medical history except for the history of using daily E-cigarettes for a duration of four years, presented with productive cough for four days and sudden onset of shortness of breath for one day. He denied any fever, chills, sore throat, chest pain, or sick contacts. His blood pressure was 128/72 mmHg, heart rate 72 beats/min, temperature 98.8-degree Fahrenheit, and oxygen saturation of 92% on 15 L/min of oxygen via face mask. Lung examination revealed diffuse crackles in bibasilar region. All other systemic examinations were unremarkable. He presented with a WBC of 15.36 mm3, with a neutrophil of 94%; arterial blood gas test revealed the following values: pH 7.47, pCO2 30.8 mmHg, pO2 41 mmHg, and bicarbonate 22.2 mEq/L. Computed tomography angiography (CTA) of lung did not show pulmonary embolism. However, it revealed extensive ground glass opacity in both lungs mostly sparing the periphery (Figure 1).

Neither cavitations were seen nor was there any evidence of bullous lung disease. Bronchoscopy revealed moderate amount of clear mucoid secretions bilaterally and diffuse bilateral mucosal hyperemia with no endobronchial lesions, masses, or foreign bodies. Bronchoalveolar lavage (BAL) sample analysis showed acute and chronic inflammatory cells with lymphocyte predominance and rare eosinophils. Cultures from BAL did not grow any organisms. Cytology did not reveal malignancy. Grocott's methamine silver stain was negative for fungi, yeast, and pneumocystis. His respiratory viral panel by polymerase chain reaction did not show viral infection; aspergillus galactomannan antigen level was 0.04 Index (reference: 0.00-0.49), beta,1-3 D glucan <31 pg/mL (reference <80), and negative hepatitis B, C and HIV serology tests. Antinuclear antibody testing was negative. Suspicion of pneumonitis secondary to vape inhalation, resulted in treatment with intravenous methyl prednisone 40 mg every 12 h for five days that was changed to oral prednisone with a plan to taper over next 28 days on discharge. His hypoxia resolved completely, and he was discharged from the hospital after seven days.

**Figure 2:**
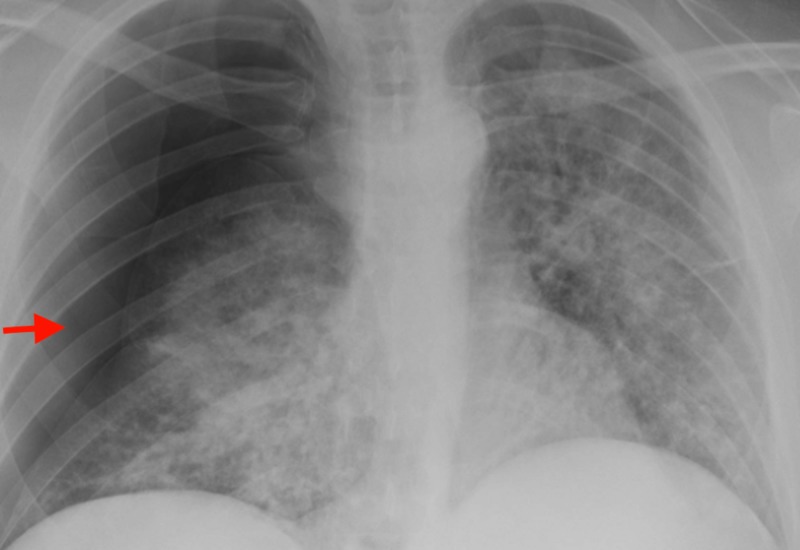
Chest X-ray showing right-sided pneumothorax with slight shift of cardiac and mediastinal structures to left.

Three days later, patient presented to the ER with a sudden onset of right-sided chest pain and dyspnea after violent bout of cough. Chest X-ray showed right-sided pneumothorax with a slight shift of cardiac and mediastinal structures to the left (Figure 2).

**Figure 3:**
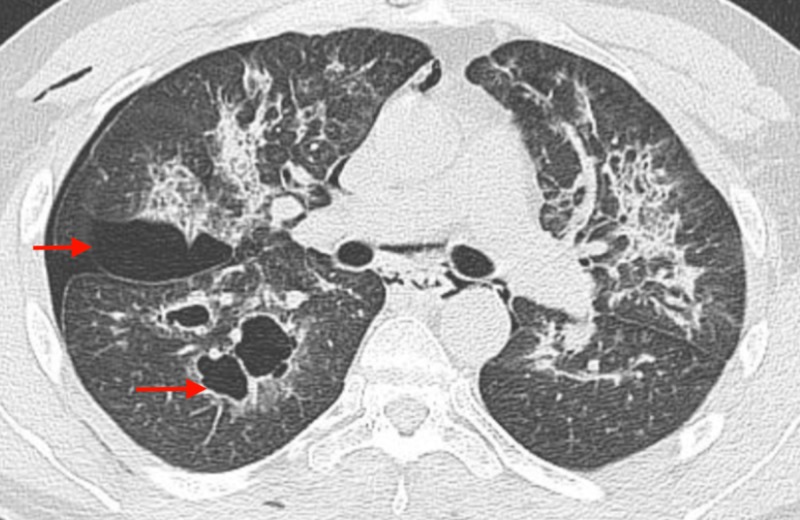
CT scan of the chest showing blebs on the right side (red arrows).

A right-sided chest tube was placed emergently. A CT scan of the chest after chest tube placement showed ground glass opacities in the central portion of lung bilaterally with new areas of cavity formed in the right upper lobe and superior segment of right lower lobe with relatively thin wall (Figure 3).

**Figure 4:**
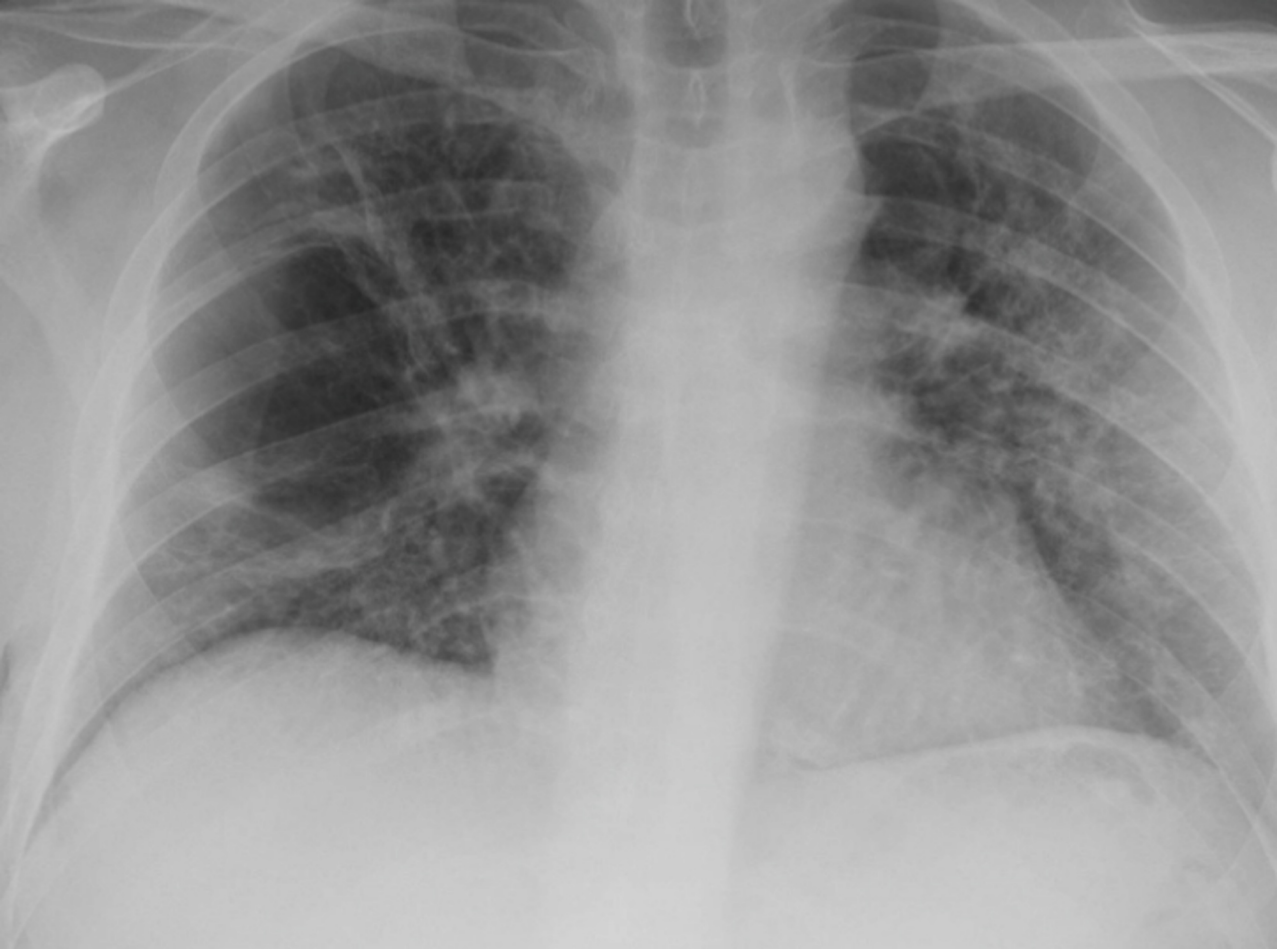
Chest X-ray showing fully re-expanded lungs after chest tube removal on right side.

The patient was treated with high-dose methyl prednisone 60 mg every six hours for three days and changed to oral prednisone 50 mg daily again with a plan to slowly taper over four weeks. His right lung fully re-expanded without any recurrence of pneumothorax even after connecting to water seal and clamping the chest tube in a stepwise manner. His chest tube was removed after four days (Figure 4).

Due to the high risk of recurrence of pneumothorax in the presence of multiple thin walled bullae on the right side, he underwent bleb removal and right-sided parietal pleurectomy through video assisted thoracic surgery (VATS). Postoperative course was unremarkable, and the patient was discharged home next day.

## Discussion

As numerous cases of E-cigarettes and associated vapor-induced lung injury begin to arise, the various pneumonitis patterns that have emerged consist of acute eosinophilic pneumonia, organizing pneumonia, acute respiratory distress syndrome (ARDS), diffuse alveolar hemorrhage, hypersensitivity pneumonitis, and the rare giant-cell interstitial pneumonitis [9]. About 80% of patients who have used vape were found to use products that contained tetrahydrocannabinol (THC) or cannabidiol (CBD) besides nicotine as the main ingredient. Contamination of such E-cigarettes with bacteria or viruses did not seem to explain the lung injury and subsequent clinical presentation mainly is pertinent to acute hypoxic respiratory failure [9]. Preliminary presentation data were recently reported on 9/6/19 by the Wisconsin Department of Health Services and the Illinois Department of Health. Fifty-three patient cases were complied with the following presentations: respiratory symptoms (98%), gastrointestinal symptoms (81%), constitutional symptoms (100%) with image findings of bilateral ground-glass opacities [10]. Additional features found were pleural effusions, pneumomediastinum, and tree-in-bud opacities. In all of these cases, there was a history of vaping that contained nicotine and THC or CBD products. In addition, a high percentage was found to have leukocytosis with a neutrophil predominance with no greater than 2% peripheral eosinophils [11].

In the current case, the patient was using mostly ‘Heavy Hitters - Cold filtering cartridges’ with a true ceramic core. It is a solvent-less formula with 89% active cannabinoids (total), 86% THC (860 mg), and 0% CBD - Sativa strain with ingredients of 'Distilled oil and terpenes.' Their website states that they do not use any thinning agents including propylene glycol (PG), vegetable glycerin, or medium chain triglycerides [12]. His history of chronic vaping and THC use and CT chest finding of bilateral diffuse ground glass pattern of parenchymal changes with sparing of periphery in absence of any other identifiable etiology led us to the diagnosis of vaping-induced pneumonitis. In the absence of definite therapy recommendations, we treated the patient with intravenous methyl prednisone initially, for hypoxemic respiratory failure and transitioned him to oral prednisone to be tapered over two weeks beyond resolution of hypoxemia. When he presented to us again with right-sided spontaneous pneumothorax, his CT scan of the chest findings showed blebs on the right side. We postulate that due to the massive inflammatory changes of the lung parenchyma caused by vapor-induced injury, he developed damage to the alveoli, resulting in thinning. Air dissected through the interstitial tissue and accumulated into the fibrous and thin layer of visceral pleura giving rise to bleb. Such subpleural blebs ruptured during bouts of vigorous coughing and gave rise to pneumothorax. To ensure prevention of a recurrent pneumothorax, a VATS procedure was performed with bleb resection and parietal pleurectomy. This is a unique case scenario as secondary pneumothorax in the context of vapor-induced lung injury has been very rarely reported before.

## Conclusions

Due to increasing use of E-cigarettes, cases of vapor-induced lung injury have been increasing lately. Our case was unique in the sense that pneumothorax due to vape/E-cigarette induced lung injury has been very rarely reported so far. Based on our experience, we recommend treating patients with diffuse vapor-induced pneumonitis with aggressive steroids with close surveillance for further complications even after resolution of initial hypoxemia.
